# Epidemiology of uveitis in children over a 10 years period

**DOI:** 10.1186/1546-0096-9-S1-P223

**Published:** 2011-09-14

**Authors:** Lydia Clarke, Yan Guex-Crosier, Michael Hofer

**Affiliations:** 1 Pediatric rheumatology Romande, DMCP, CHUV, Lausanne and HUG, Genève, Switzerland; 2 Immuno-infectiology Eye Clinics, Hôpital Ophtalmique Jules Gonin, Lausanne, Switzerland

## Introduction

Uveitis is an inflammation of the vascular tunic of the eye (uvea), usually idiopathic, infectious or related to a rheumatologic disease. In children it is a responsible for more than 17% of unilateral legal blindness.

## Purpose

To investigate the distribution, clinical features, complications and loss of visual acuity in paediatric uveitis in the French speaking part of Switzerland treated according to a multidisciplinary approach.

## Methods

Retrospective cohort study of all patients diagnosed with uveitis under the age of 16 years presenting to two tertiary referral centres in Lausanne (uveitis and paediatric rheumatology clinics), between 2000 and 2009.

## Results

79 children (37 girls) were identified, 62 lived in Switzerland, 11 in Italy. Mean age at first symptoms was 9.0 years (1.5-15.8 years) with a mean follow-up time of 1.9 years (0-8 years). 51 had involvement of both eyes (64.6%). The course was acute in 26.5%, chronic in 51.9%, recurrent in 8.9% and not specified in 12.7%. Anterior uveitis occurred in 40.5%, intermediate in 32.9%, posterior in 25.3% and panuveitis in 6.3%. The three main diagnoses were idiopathic uveitis (34.2%), juvenile idiopathic arthritis-related (22.8%) and toxoplasmosis (15.2%). More than the half of the patients was treated with systemic therapies: corticosteroids 39, methotrexate 17, azathioprine 11, cyclosporine 4 and biotherapies 13. The visual acuity of most patients remained stable (37.5%), or improved (36.3%) during the follow-up. Only 7.5% eyes had a loss of visual acuity (one or more lines) during the follow-up. 26.3% patients presented one or more ocular complications. The two commonest were cataract (12.5%) and ocular hypertension (10%).

## Conclusion

Our patients show a better visual outcome than previously published cohorts. Thus, early recognition and prompt and aggressive treatment are essential to reduce the rate of complications and improve the visual outcome. Collaboration between ophthalmologists and rheumatologists enables a better management of systemic treatments and prompt access to immunosuppressive agents and biotherapies.

